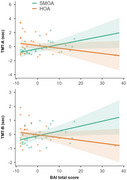# Influences of general and specific psycho‐social factors on cognitive health of sexual minority older adults

**DOI:** 10.1002/alz70857_102642

**Published:** 2025-12-25

**Authors:** Riccardo Manca, Annalena Venneri

**Affiliations:** ^1^ Brunel University London, Uxbridge, Middlesex, United Kingdom; ^2^ University of Parma, Parma, Italy; ^3^ Brunel University London, London, United Kingdom; ^4^ University of Parma, Parma, Italy, Italy

## Abstract

**Background:**

Sexual minorities (i.e. people who identify as non‐heterosexual) show several health disparities. However, risk/protective factors relevant to cognitive health of sexual minority older adults (SMOAs) have not been fully investigated. The aim of this study was to quantify the impact of several psycho‐social factors on subjective and objective cognitive health in SMOAs.

**Method:**

Thirty‐nine cognitively unimpaired SMOAs (age = 64.1±10.0; M = 74.4%) and 30 heterosexual older adults (HOAs; age = 65.6±9.7; M = 40.0%) completed an online survey to provide information on psycho‐social risks (i.e., depression, anxiety, general stress), subjective cognitive decline (SCD), beliefs about their memory and dementia worries. SMOAs also completed questionnaires on minority stress and outness. Twenty‐seven SMOAs and 24 HOAs underwent a comprehensive neuropsychological battery. General linear models were used to assess the relationship between psycho‐social risk factors and cognitive health and the interactions between sexual orientation and risk factors. Minority stress and outness were also investigated as risk/protective factors for cognitive health in SMOAs only.

**Result:**

Compared with HOAs, SMOAs reported worse mental and subjective cognitive health (i.e. SCD for memory, negative memory beliefs and dementia worries), but similar cognitive performance. Worse depression/anxiety were associated with more dementia worries in both groups, while anxiety was associated with worse performance on the Trail Making Test in SMOAs only (Figure 1). Moreover, worse minority stress was associated with higher risk of SCD for language (OR = 6.79, *p* =  0.048) and more negative memory beliefs (b = 0.41, *p* =  0.012), while being more out about one's own sexual orientation was associated with better short‐term memory performance (b = 0.54, *p* =  0.004).

**Conclusion:**

This is the first targeted study highlighting that SMOAs experience more detrimental effects of anxiety than HOAs on attentional functions. Minority stress primarily affects subjective cognitive health of SMOAs, while outness appears to provide resilience against cognitive decline. Further investigations are needed to ascertain longitudinal cognitive changes and to devise potential prevention strategies aimed at preserving cognitive health in SMOAs.